# Excipients Used for Modified Nasal Drug Delivery: A Mini-Review of the Recent Advances

**DOI:** 10.3390/ma15196547

**Published:** 2022-09-21

**Authors:** Chrystalla Protopapa, Angeliki Siamidi, Panagoula Pavlou, Marilena Vlachou

**Affiliations:** 1 Department of Pharmacy, Section of Pharmaceutical Technology, School of Health Sciences, National and Kapodistrian University of Athens, 15784 Athens, Greece; 2 Department of Biomedical Sciences, Division of Aesthetics and Cosmetic Science, University of West Attica, 28 Ag. Spyridonos Str., 12243 Egaleo, Greece

**Keywords:** nasal drug delivery, poloxamer, chitosan, gellan gum, vaccines, excipients, nanoparticles

## Abstract

The ongoing challenging task in the field of nasal drug delivery is the maintenance of an efficient concentration of the active substance in the target area for an adequate period of time. Thus, there is an urgent need to develop effective new strategies for drug delivery to the nose, using cutting edge technology and materials for this particular type of drug delivery. This review gives an account of the critical components of nasal drug delivery and the parameters influencing drug absorption in the nose, including the excipients required for modified drug administration.

## 1. Introduction

**Figure 1:**
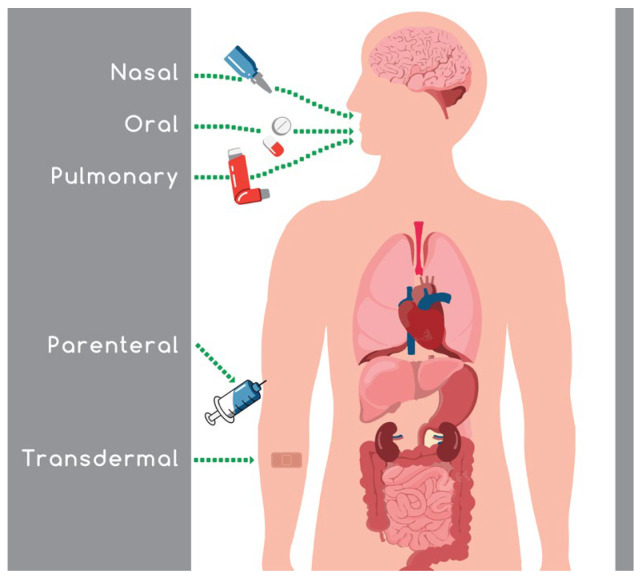
Different routes of drug administration.

Oral administration of medicines is the most convenient method of drug dispensing with the highest compliance rates, as patients can self-administer their medications in a noninvasive and painless manner, providing a variable and customizable dosing schedule at home [1]. On the other hand, the harsh chemical environment in the stomach and intestines, the first-pass effect, and the barrier of the gastrointestinal tract pose major difficulties for the efficacy of drugs [2,3]. Thus, alternative routes of administration have been developed to circumvent those hurdles and achieve the requisite results (Figure 1). The major alternative routes are pulmonary, transdermal, buccal, nasal, and parenteral [2], and each of these has its own advantages and disadvantages, as shown in Table 1.

Intranasal absorption is a favored route because it avoids the gastrointestinal and hepatic metabolism, leading to an increase in drug bioavailability, and a reduction in the side effects and the required dose administered [4]. Furthermore, the intranasal route also has several practical advantages for both the patient and the pharmaceutical industry. The former can benefit from the non-invasive, highly tolerable, easy to administer, and pain-free drug delivery systems, and pharma avoids the need to sterilize the nasal products [5]. Moreover, acid-sensitive drugs, such as biologics (peptides, hormones, etc.), which are prone to degradation in the gastrointestinal tract, and drugs that cannot be absorbed orally, may be administered nasally. The inclusion of enhancers, mainly excipients, can increase the nasal absorption of such drugs [6]. Recently, many pharmaceutical companies have become involved in the commercialization of absorption promoters and absorption modulators for nasal drug delivery [7].

Even though the nasal epithelium acts as a tight barrier, the intercellular junctional complex of the nasal mucosa is not impervious, due to the leaky epithelial tissue [8,9]. Thus, in combination with the high vascularization of the nasal surface, a favorable profile for therapeutic purposes, more than just the topical nasal drug delivery is achieved [10,11].

Recent developments in the field of dosage forms have produced numerous novel drug delivery systems that play an important role in improving therapeutic efficacy. The aim of this review is to summarize the recent advances—during the past 5 years (2017–2022)—in excipients used in semi-solid formulations and vaccines used for modified nasal drug delivery to help formulation scientists select the optimal excipients for nasal product manufacturing.

## 2. Anatomy and Physiology of the Nose

The primary roles of the nasal cavity are olfaction and breathing [12]. Nonetheless, the breathing air is filtered, humidified, and headed by the nasal cavity before reaching the lungs, whilst the inhaled particles and pathogens are trapped from the hair and the mucus layer present in the nasal cavity. Other functions of the nasal structure are the metabolism of endogenous substances and the immunological activities [13,14]. 

**Figure 2:**
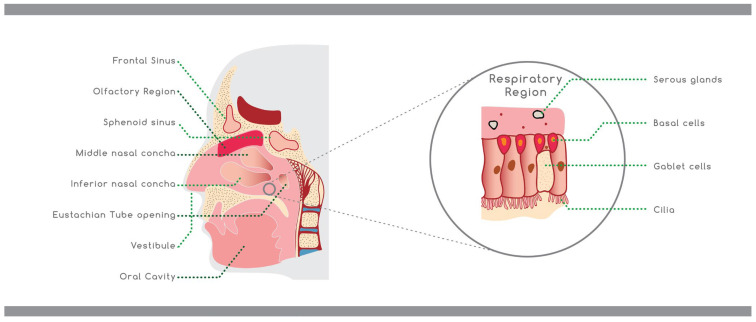
Schematic representation of the nasal cavity.

The nasal cavity is located between the roof of the mouth and the base of the skull supported from above by the ethmoid bones and from the side by the ethmoid, maxillary, and inferior conchae bones. The entire surface area is almost 150 cm^2^ and has a total volume of 15–20 mL [11,15]. The nasal cavity consist of three definite parts: the vestibule, the respiratory, and the olfactory regions (Figure 2) [16]. The anterior part of the nasal cavity is the nasal vestibule, which is part of the nostrils, covering an area of about 0.6 cm^2^, and includes the nasal hairs (vibrissae) [17]. Histologically this part is covered by a keratinized and stratified squamous epithelium with sebaceous glands [10]. These nasal regions prevent the insertion of toxic materials, but at the same time, drug absorption is also limited [18]. The respiratory region represents the largest part of the nasal cavity. It is split into three turbinates—the superior, middle, and inferior—which are responsible for the humidification and temperature adjustment of the inhaled air [19].

The most important area for systemic drug delivery is the nasal respiratory mucosa, which consists of the epithelium, the basement membrane, and the lamina propria. The epithelium contains the pseudostratified columnar epithelial cells, goblet cells, basal cells, and mucous and serous gland cells. Many of the epithelial cells are coated by microvilli, which enhance the respiratory surface area, and the cilia, which are fine projections essential for mucus transport towards the nasopharynx [20]. The secretory glands and the goblet cells produce mucus through granules filled with mucin, a glycoprotein that define the mucus viscosity. The mucus is deposited as a thin layer (about 5 μm) in the epithelium, composed of water (95%), 2.5–3% of mucin, and 2% of other substances, such as electrolytes, proteins, enzymes, lipids, antibodies, sloughed epithelial cells, and bacterial products [21]. It is responsible for the humidification and warming of the inhaled air, it has a pH between 5–6.5, and it provides protection to the nasal epithelium against foreign particles and drugs [22,23].

The olfactory region is located on the top of the nasal cavity and continues down the septum and the lateral wall. The olfactory epithelium is pseudostratified and consists of specialized olfactory receptor cells essential for smell recognition [24]. 

## 3. Nasal Drug Delivery

The nasal route of administration is used for treating local inflammation, allergic and common rhinitis, and nasal congestion. The active compounds that are commonly used against these diseases are antihistamines, glucocorticoids, or decongestants in the form of nasal spray, drops, solutions, gels, or powders and other types of formulations, including emulsions, suspensions, and microparticles [6,25]. The nasal route is used for either local or systemic action. The drug is administered locally for rapid alleviation of the symptoms of the disease, reducing the administered dose, as the drug is placed directly in the affected area, thus avoiding the systemic metabolism. On the other hand, the nasal administration of pharmacologically active substances for systemic action is used in the case of drugs with poor intestinal absorption and limited stability in the gastrointestinal fluids, with extensive hepatic first-pass metabolism, such as biologics and polar drugs [10,11]. The administration of the drug through the nasal passages can also bypass the blood–brain barrier (BBB), so it can be used for central nervous system (CNS) action. This route has been further studied for administrating vaccines [11,26]. 

The drug absorption through the nose is based on the physicochemical properties of the administered drug. The drug cannot penetrate the mucosa and manifest its action if it has a large size (greater than 1 kDa), a high degree of ionization, or is too lipophilic. Another factor that can affect absorption is the drug’s pH, which may affect the stability and the ionization of the drug, as well as cause nasal irritation. Formulations that have high viscosity can more easily enter the nasal mucosa, but simultaneous, they may be less absorbed. When the hyper- or hypotonicity is very high, the ciliary movement can be altered, resulting in lower absorption [6]. Finally, the drug’s concentration and quantity, the position of the head during administration, the nasal surface, and the physical condition of the dosage form all play a vital role in the absorption of the drug [27]. 

## 4. Factors That Affect the Nasal Drug Absorption

Drug delivery through the nasal route of administration has some limitations, which are crucial because they influence drug concentration and bioavailability and therefore, the absorption and the pharmacological effect of the administered drug. The first main barrier is the range of pathological and physiological conditions linked to the nasal mucosa, which can affect the absorption and efficacy of the drug [28]. For example, a physiological change in nasal mucosa based on illness and allergy (irritation and the inflammation of the nasal cavity, which is intensified by itching and sneezing) may influence drug absorption [6]. 

In addition, there is a restriction regarding the absorption of the poorly water-soluble drugs due to the low volume of the nasal cavity, which reduces the administered amount to 100–150 μL [28]. The permeability also decreases for the polar and large molecules and for peptides and proteins [25]. However, by using the correct excipients, including bioadhesive polymers, enhancers, and enzymatic inhibitors, the drug permeability and residency in the nasal cavity can be improved [29].

Another crucial barrier is the mucociliary clearance (MCC) of the mucosa, which by replacing the mucus layer every ~15 min with 5–6 mm/min, the transmucosal absorption is decreased. The mucus can also decrease the drug absorption by binding the drug to mucin, which is the primary protein of the mucus. While the small moieties can pass through easily, the charged or larger units can be caught in the mucus gel [25]. Mucus also contains different enzymes which can influence the stability of protein- and peptide-based drugs; proteases degrade peptides and proteins by attacking them. These xenobiotic enzymes [e.g., P450 monooxygenase, Phase I enzymes (flavin monooxygenases, aldehyde dehydrogenases, epoxide hydrolases, carboxylesterases, etc.) and Phase II enzymes (glucuronyl and sulphate transferases, glutathione transferase) can also metabolize intranasally administered small-molecule drugs, such as opioids, histamines, corticosteroids, etc. [30]. Moreover, chemical bonds are created between the protein drugs and immunoglobulins, leading to bulky molecules that are no longer able to penetrate the nasal mucosa [31]. After permeating the mucus layer, the main process of drug penetration through the mucosa combines transcellular and paracellular passive diffusion and transcytosis by the vesicle carriers [32,33].

Last but not least, there will always be concerns about the safety of nasal medicines, even though recent breakthroughs in both in vitro and in vivo models are a major benefit in speeding up clinical development and eventually, the time-to-market of new treatments [33].

## 5. Excipients Used in Modified Drug Release Semi-Solid Pharmaceutical Dosage Forms for Nasal Administration

**Figure 3:**
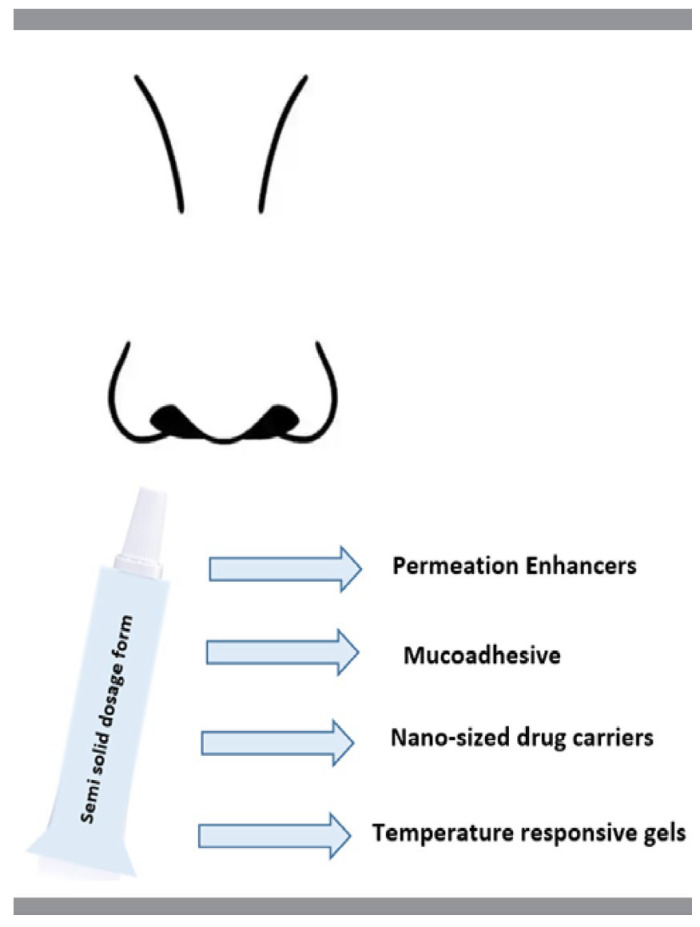
Different semi-solid formulations for nasal administration.

To improve nose-to-brain drug transfer and to extend drug residence time in the nasal cavity, several approaches could be employed, such as the use of semi-solid dosage forms, permeation enhancers, mucoadhesive and temperature responsive gels, or nano-sized drug carriers (Figure 3). In this section, the excipients and methods used in the recent achievements (2017–2022) in the modified release semi-solid formulations for nasal administration are reviewed and summarized, as shown in Table 2.

### 5.1. Poloxamers

Thermoresponsive hydrogels composed of poloxamers are of great interest for pharmaceutical applications, particularly for ocular, nasal, injectable, transdermal, and vaginal medication delivery. These hydrogels exist in liquid form at room temperature, but turn into a more viscous gel when they come in contact with body temperature. Therefore, the gelling system remains in place for a longer period of time, and the release of the active ingredient is slowed. Poloxamers are synthetic triblock copolymers of poly(ethylene oxide)-b-poly(propylene oxide)-b-poly(ethylene oxide) (PEO-PPO-PEO) [34]. Due to their amphiphilic character, they exhibit the characteristics of a surfactant that can interact with hydrophobic surfaces and biological membranes. In aqueous media, above the critical micelle concentration, they independently form micelles (10–100 nm). The core of the micelles consists of hydrophobic PPO segments separated from the aqueous surface by a hydrated shell of hydrophilic PEO blocks. The core of these micelles can be used for the uptake of various therapeutic/diagnostic chemicals and for transporting water-insoluble drugs [35], due to PPO’s hydrophobic nature. However, it has been reported that its parenteral administration may lead to serious alterations in lipid metabolism and renal filtration, as observed in animal models where a high intra-peritoneal dosage was used; thus, long-term use should be avoided [36]. 

Poloxamers have recently been widely used for the development of in situ nasal gels, which have mucoadhesive and thermoreversible properties. Compared to liquid nasal preparations, in situ nasal gels are instilled into the nasal cavity as low-viscosity solutions. When in contact with the nasal mucosa, the thermosensitive polymer changes its conformation and forms a gel. As a result, it can not only extend the contact duration between the medication and the receptive areas in the nasal cavity, but also release the drug at a slower pace. The included drug is usually released by diffusion through the gel matrix. In a recent work, the combination of poloxamers 407, 188, and chitosan was studied in huperzine A formulations. The results showed a biphasic release behavior, with an initial quick release during the first 30 min, followed by a sustained release over a 24 h period. It was found that the combination of poloxamers and chitosan improved flowability and mucoadhesion, which prolonged the drug residence time [37]. In another study, researchers used poloxamers 407, 188, and Na-CMC, along with almotriptan. This combination also exhibited a biphasic release pattern, with an initial quick release within the first 30 min (anti-migraine effect of the drug for the management of acute migraine pain), followed by a sustained release over a 5 h period (required to maintain the loading dose) [38].

These two poloxamers (407 and 188) were also used in combination with carrageenan to form a novel in situ intranasal gel with mucoadhesive properties. The release of the drug sumatriptan followed a biphasic profile. Release data analysis proved that the diffusion-driven mechanism was the predominant driving force, following first-order kinetics [39]. In another work, scientists prepared an in situ nasal gel of ziprasidone-*β*-cyclodextrin to improve bioavailability, because the cyclodextrin can improve the solubility of the drug by modifying its physicochemical properties. Poloxamers (407 and 188) were selected as gelling agents; polyethylene oxide was also used to induce gelation and HPMC K4M to improve mucoadhesive strength. The results indicated significant release and mucoadhesion that confirmed adequate residence time at the site of action [40]. For a longer residence time in the nasal cavity, which could lead to increased bioavailability of geniposide, researchers formulated an in situ gel using poloxamers (P407, P188) and HPMC. The combined use of these polymers and HPMC indicated a controlled release of the drug through corrosion of the gel [41].

Mucoadhesive thermoreversible in situ nasal gels have also been formulated with poloxamer 407, in combination with various polymers. Researchers have developed polymeric nanoparticles (NPs) to increase diffusion through mucus via the nasal route for nose-to-brain drug delivery. NPs loaded with rivastigmine hydrogen tartrate in poloxamer 407 resulted in prolonged and sustained drug release over a 24 h period [42]. In another study, scientists used poloxamer 407 and Carbopol^®^ 974P NF to develop in situ gelation-sensitive hydrogels for the sustained release of mometasone furoate. The delayed drug release was attributed to the viscosity-increasing effect of the mucoadhesive polymers and the squeezing effect on the aqueous channels of the poloxamer micelles through which the drug diffuses [43]. In addition, poloxamer 407 was used in combination with HPMC K4M to control the release properties of montelukast sodium, with good results [44]. Moreover, poloxamer 188 was used in combination with Carbopol 934 for intranasal delivery of locally acting drugs, such as hydrocortisone. A longer residence time in the nasal cavity is required to achieve a prolonged effect. These two polymers exhibit improved mucoadhesive properties by forming an in situ gel that could provide a longer residence time for the treatment of allergic rhinitis [45].

**Table 2:** Semi-solid formulations for modified release nasal drug delivery.

Nasal Dosage Form	Drug Release Rate *	API	Excipients	Refs.
in situ gel	biphasic	huperzine A	poloxamers (407, 188), CS, castor oil, polyoxyl 40 hydrogenated castor oil, 1,2- propanediol, Ringer’s solution	[37]
in situ gel	biphasic	almotriptan	poloxamer (407, 188), Na-CMC, glyceryl behenate glyceryl palmitostearate, glyceryl monostearate, precirol	[38]
in situ gel	biphasic	sumatriptan	poloxamers (407, 188), carrageenan, soybean phospholipids, cholesterol, tween 80, sodium caprate, sodium cholate, clostridium perfringens enterotoxin, sodium caprate	[39]
in situ gel	controlled	ziprasidone	poloxamers (407, 188) *β*-cyclodextrin, HPMC E5, PEG 6000, PEG 4000, polyethylene, HPMCK4M	[40]
in situ gel	controlled	geniposide	poloxamers (407, 188), HPMC, borneol, benzalkonium chloride, NaCl	[41]
in situ gel	sustained	rivastigmine hydrogen tartrate	poloxamer 407, poly (lactic-*co*-glycolic acid), polymeric NPs	[42]
in situ gel	sustained	mometasone furoate	poloxamer 407, Carbopol^®^ 974P NF, PEG 400, NaCl, benzalkonium chloride, dexpanthenol, triethanolamine	[43]
in situ gel	controlled	montelukast sodium	poloxamer 407, HPMC K4M, PEG 400, methyl paraben	[44]
in situ gel	controlled	hydrocortisone	poloxamer 188, Carbopol 934, PG, benzalkonium chloride, triethanolamine, isopropyl alcohol	[45]
NP	biphasic	pramipexole dihydrochloride	CS, sodium tripolyphosphate	[46]
NP	biphasic	efavirenz	CS chloral hydrate, glucosamine chloral hydrate, *N*-acetylglucosamine, HP-*β*-CD, Tween 80	[47]
NP	controlled	sitagliptin	CS, glacial acetic acid, tripolyphosphate	[48]
NP	delayed	human serum albumin	CS low molecular weight, acetic acid, mucin, sialic acid	[49]
in situ misemgel	controlled	raloxifene hydrochloride	peppermint oil, *n*-propanolol, *n*-butanol, Tween^®^ 80, PEG 200, PG, GG, TPGS, linoleic acid, Kolliphor^®^, RH 40	[50]
in situ gel loaded NPs	biphasic	voriconazole	GG, clove oil, nanotransferosomes, Tween 80, lecithin	[51]
nanoemulsion	biphasic	quetiapine	Capmul MCM, Emalex LWIS 10, PEG 400, Transcutol P, Tween 80, water, Labrafil M 1944 CC, isopropyl myristate, sesame oil, Lauroglycol 90, miglyol 840	[52]
NPs	sustained	dolutegravir sodium	HP-*β*-CD, DPC, Tween 80, DMSO	[53]
NPs	slow	acetylcholinesterase reactivator	*L*-α-phosphatidylcholine, 75% soybean phosphatidylcholine, dihexadecylmethylhydroxyethylammonium bromide, Tween 80, Phospholipon 80, Lipoid S75, 1-(*o*-tolylazo)-2-naphthol, pyrene, pyridine-2-aldoxime methochloride (Pralidoxime)	[54]

* Drug release rate as stated by the author(s); CS: chitosan, DPC: diphenyl carbonate, GG: gellan gum, HPMC: hydroxypropylmethylcellulose, HP-β-CD: hydroxypropyl-β-cyclodextrin, Na-CMC: sodium carboxymethylcellulose, NPs: nanoparticles, PEG: polyethylene glycol, PG: propylene glycol, TPGS: d-α-tocopheryl polyethylene glycol 1000 succinate.

### 5.2. Chitosan

To enhance nasal absorption, chitosan (CS) has also been utilized due to its biocompatibility, biodegradability, low toxicity, and its ability to modulate drug release. Because the nasal cavity is prone to ciliary clearance, its mucoadhesive characteristic is very relevant in nasal administration systems. Ionic interactions have been discovered between positively charged CS amino groups and negatively charged sialic acid groups of mucin or other negatively charged nasal mucosa groups [55]. Due to these characteristics, CS is a desirable material for NP [56]. Researchers have used CS in the preparation of NPs in a pramipexole dihydrochloride formulation. The results indicated biphasic release; the initial fast release may be attributed to the drug’s release from the NP surface, while the prolonged diffusion seen at subsequent time points may be due to drug release from the NPs’ core owing to hydration and swelling. [46]. Another group of researchers used CS to formulate intranasal CS-g-HP-*β*-CD NPs to enhance the solubility of the lipophilic drug Efavirenz. The release profile was characterized as biphasic for the same reasons [47]. Controlled drug release was detected in sitagliptin CS-NPs. As CS is a swellable polymer, the initial drug release was mediated by diffusion across the polymer, followed by polymer matrix relaxation [48]. Drug release modulation was also noticed when NPs with a CS coating were used [43].

### 5.3. Gellan Gum

Gellan gum (GG) is a polymer used in the pharmaceutical industry as a tablet binder, disintegrant, gelling agent, and a controlled release polymer. In a recent study, an in situ misemgel for a nasal drug delivery was produced with GG, exhibiting the controlled release of Raloxifen, with high drug permeation. In terms of micelles, *d*-α-tocopheryl polyethylene glycol 1000 succinate (TPGS) might boost paracellular absorption by momentarily disrupting cell organization; moreover, nanosized self-emulsifying systems could improve transcellular absorption via passive diffusion due to lipid composition [50]. In another study, nanotransferosomes were loaded into an in situ gel system for the biphasic release of voriconazole. Initial incomplete gel formation caused drug burst release, but in subsequent stages, gelation occurred and slower drug release was observed [51].

### 5.4. Nanosized Drug Carriers

In general, nanoscale drug delivery systems concentrate on the formulation of bioactive compounds in biocompatible nanosystems. These engineered systems are usually targeted to a specific site or are intended for modified drug release. In some cases, the nanoparticulate drug delivery systems exhibit several types of toxicities, i.e., cellular toxicity, genotoxicity, long term toxicity, etc. The physicochemical characteristics of these systems, their composition, their shape, and their surface charge are the critical parameters that influence their nanotoxicity. Specifically, the vesicular drug delivery systems are more biocompatible in comparison to rod-like shaped systems. Additionally, the cationic charge is responsible for immunogenic and allergic reactions in comparison to neutral or negative drug delivery platforms [57]. The size below 100 nm is identical for the interactions of these systems with subcellular organelles. The last phenomenon could sometimes lead to unexpected side effects. According to the regulations and the regulatory guidelines, the IC_50_ should be determined and addressed for drug delivery system [58].

Several attempts have been made to use nanosized drug carriers in nasal drug delivery. In particular, a biphasic release pattern was observed in a quetiapine nanoemulsion formulation. Initially, a rapid drug release was observed, attributed to micellar solubilized active moiety that causes an initial burst release, followed by a slower release pattern credited to the drug release from oil droplets [52]. In another study, researchers produced NP with dolutegravir sodium. The sustained drug release observed could possibly be attributed to the cross-linked polymer network formed during the nanoprecipitation process, which caused the entrapped drug to diffuse slowly from the nanostructure. High brain–drug transport percentage was also observed [53]. Moreover, a group of scientists used cationic liposomes to penetrate the nasal mucus, and using acetylcholinesterase reactivator as a model drug, they probed its release from the nanoparticles. Drug release was noticed during the last three hours, three-fold slower than in the case of an aqueous solution [54].

## 6. Excipients Used in Modified Drug Release Vaccines for Nasal Administration

The majority of current vaccinations are designed to be administered systemically by intravenous, intramuscular, subcutaneous, or intradermal routes. Parenterally given intranasal vaccination formulations increase cellular and humoral protection against infections. Because the mucosal and systemic immune systems work differently, the parenteral vaccine cannot make contact with a mucosal barrier to induce mucosal immunity. Vaccine delivery through the nasal route, on the other hand, induces systemic immunity, as well as a powerful mucosal immune response. As a result, nasal administration can be used to deliver proteins, and peptides, as well as medicines. Various carriers of different systems for nasal vaccine delivery include powders, gels, and solutions [59]. In this section, the excipients and methods used in the recent developments (2017–2022) in the modified release intranasal vaccines are reviewed and summarized, as shown in Table 3.

### 6.1. Chitosan

As previously mentioned, CS is a biodegradable, biocompatible, non-toxic, and hydrophilic cationic polysaccharide generated chemically from the partial deacetylation of chitin. Because they may improve membrane permeability and bioavailability of macromolecules, CS NPs have potential uses in the modified release of drug and antigen delivery systems. According to immunohistologic studies, CS can untie epithelial cell tight junctions, allowing cells to pass the mucosal barrier. To improve solubility, enhance mucoadhesiveness, and increase the adjuvant effect, CS derivatives, such as trimethyl CS and glycol CS, have also been formulated. In a research work, a new derivative of CS—CS maleimide—was synthesized and used in the formulation of NPs as a novel intranasal vaccine delivery system, with prolonged retention time on the mucosa [60]. In other studies, in order to enhance vaccine efficacy, to protect the DNA from nasal mucosa degradation, and also to promote a slow antigen release at the targeted site of vaccination, scientists have used CS NPs with LACK-DNA plasmid [61] or aminated and aminated plus thiolated CS NPs with bovine serum albumin [62]. CS was also used as a delivery carrier, along with NPs, and bovine serum albumin, for administration into the lungs of patients affected by COVID-19. NPs’ physiochemical properties can be modified with CS, leading to an increase in the dispersibility of particles, culminating in high deposition into the lungs [63]. Moreover, CS as a mucoadhesive polymer can adhere to and permeate the mucosa of lung epithelial cells and the wide intercellular compact junctions of the lung’s epithelium [64]. CS has also been used in conjunction with poloxamers (188 and 407) for the preparation of a CS-hydrogel vaccine against influenza virus infection. The incorporation into the gel showed prolonged appearance of the antigen within the nasal tissue of the upper respiratory tract [65]. For the biphasic release of antigens, CS and its derivatives have also been studied. Specifically, the release of r4M2e.HSP70c and r4M2e from trimethyl CS NPs was 65% within the first 12 h, followed by moderate release over the next 48 h (about 80%), and total release up to 93% after 100 h [66]. In another work, the nasal release tests of tetanus toxoid from trimethyl CS NPs showed a burst release within 1 h, followed by a plateau release up at to 4 h [67]. The same model antigen of tetanus toxoid was also encapsulated with CS NPs. The results indicated a burst release of ~40% in the first 30 min, followed by a sustained release [68]. In another study, bovine serum albumin, ovalbumin, and myoglobin were used as model antigens for NPs loading. Low molecular weight CS was used for the gradual release of the antigens [69].

**Table 3:** Vaccines for modified release intranasal immunization.

Vaccine	Release Rate *	API	Excipients	Refs.
NPs	extended	Encephalitis-chimeric virus	trimethyl CS, glycol CS, 6-maleimidohexanoic acid, 1-ethyl-3-(3-dimethylamino propyl)carbodiimide, *N*-hydroxysuccinimide, sodium tripolyphosphate, phenylmethylsulphonyl fluoride, fluorescein isothiocyanate-conjugated bovine serum albumin, bovine serum albumin, polystyrene microplates, IFN-γ, IL-4 cytokine	[60]
NPs	slow	plasmid DNA encoding 5p36/LACK leishmanial antigen	CS microparticles, glyceraldehyde	[61]
NPs	controlled	bovine serum albumin	aminated CS, aminated and thiolated CS, CS, *N*-(2-hydroxyethyl) ethylenediamine, *N*-(3-dimethylaminopropyl)-*N*′-ethylcarbodiimide hydrochloride, thioglycolic acid, (3-(4,5-dimethylthiazol-2-yl)-2,5-diphenyltetrazolium bromide), trypsin-EDTA	[62]
hydrogel	prolonged	antigen that generates nasal tissue resident memory CD8+ T cells	CS, poloxamers (188 and 407), ovalbumin protein, lipopolysaccharide	[65]
NPs	biphasic	r4M2e.HSP70c antigen	*N*,*N*,*N*-trimethyl CS, trimethyl CS, glycerin	[66]
NPs	biphasic	tetanus toxoid	CS, NPs, paraffin oil, nanospheres	[67]
NPs	biphasic	tetanus toxoid	*N*-trimethyl CS, CS, dextran microspheres, tripolyphosphate, lactose, Span 80, Tween 80	[68]
NP	gradual	bovine serum albumin, ovalbumin, and myoglobin	low molecular weight CS, Compound 48/80, MTT (3-[4, 5-dimethylthiazol-2-yl]-2,5-diphenyl tetrazolium bromide), albumin-fluorescein isothiocyanate conjugate (FITC-BSA), trehalose, Dulbecco’s modified Eagle medium (DMEM) and Roswell Park Memorial Institute (RPMI), Bicinchoninic acid (BCA) assay and micro BCA kits, Fetal bovine serum (FBS), wheat germ agglutinin Alexa Fluor^®^ 350 Conjugate and Lysotracker^®^ Red DND 99	[69]
NPs	extended	PPE17 antigen (for tuberculosis)	CS, SA	[70]
NP	burst release prevented	PR8 influenza virus	SA, CS, *N*,*N*,*N*-trimethyl CS, concanavalin A	[71]
NPs	biphasic	inactivated influenza virus	SA powder, class B CpG ODN 2007 with a phosphorothioated backbone, 2,3-bis-(2-methoxy-4-nitro-5- sulfophenyl)-2*H* -tetra- zolium-5-carboxanilide, Tween 80 and Span 80	[72]
NPs	prolonged	bovine serum albumin	Poly(*D*,*L*-lactide-co-glycolide), Bisphenol-A-ethoxylate di-acrylate, ethylenediamine, tetrahydrofuran, poly(vinyl alcohol)	[73]
nanogel	gradually	surface protein A fusion antigens	pullulan with 1.3% cholesterol and 23% amino residues	[74]
nanogels	complete release in 6 h	Ovalbumin	squalane oil, cyclohexane, surfactant sucrose laurate (L-195)	[75]
nanodispersion	prolonged	Ovalbumin	Epsiliseen^®^-H (*ϵ*-polylysine), dextran sulfate sodium salt, hydrogen chloride, sodium hydroxide	[76]

* Drug release rate as stated by the author(s); CS: chitosan, NPs: nanoparticles, SA: sodium alginate.

### 6.2. Sodium Alginate

Sodium alginate (SA) hydrogels have a wide range of applications in the modified release of drugs due to their unique gelation properties that retard drug release. In the case of vaccination, in vitro release studies have shown that NPs coated with SA follow an extended antigen release pattern [70,71]. This negatively charged polymer, SA, can be used to coat the opposite (positively) charged CS or TMC, modifying the immunostimulatory properties of the NPs, increasing their stability and preventing the burst release of the loaded antigens, modifying their release behavior [71]. In another study, SA NPs were synthesized and inactivated influenza virus was incorporated into them. The antigen showed an initial burst release, followed by a plateau phase [72].

### 6.3. Nanosized Drug Carriers

Nanoparticles have been found to be excellent adjuvants and drug delivery systems for vaccine formulation, as they offer several advantages, including enhanced antigen uptake, protection from enzymatic degradation, the depot effect (gradual release of the antigen, which leads to stronger and longer lasting immune responses), a sustained release of the antigen in the mucosa, and the co-delivery of antigens and adjuvants to the same cell population [69].

Indeed, NPs prepared by PbAE and PLGA showed a prolonged antigen release [73]. In another study, researchers studied a novel pneumococcal vaccine nanogel formulation, where the antigen gradually released from the nanogel substrate into the nasal epithelial cells, even 12 h after administration [74]. Ovalbumin has been used in nanosystems in squalane oil, as its hydrogenated form has been favored due to its outstanding chemical stability, resistance to oxidation, and metabolizing characteristics. The aim of the scientists involved in this study was the delivery of antigens to the immune system from a controlled release formulation (43% antigen release within 72 h) to maximize its exposure to the immune system. Results indicated that the nanogel released the antigen content via simple diffusion through the swollen network of the polymer [75]. In order to overcome problems related to oil in water emulsions (i.e., the short duration of antigen retention in the nasal cavity due to the low viscosity of water-based emulsions, low antigen loading efficiency because antigens are simply mixed with oil-droplets rather than encapsulated inside oil, and bursting or incomplete release, which causes only a portion of the antigen to be transported to the immune system), researchers formulated an intranasal vaccine with solid-in-oil nanodispersions. Again using ovalbumin and various oils, an optimal nanodispersion formulation was produced which extended the time that the antigen remained intranasally [76].

## 7. Conclusions

In conclusion, for dosage forms aimed at the nasal cavity, the use of permeation enhancers, mucoadhesive and temperature responsive gels, or nano-sized drug carriers is important in order to achieve extended residence time in the nasal area, allowing for better drug diffusion across the nasal mucosa. Poloxamers, CS, GG, and nanoscale drug delivery systems are formulants/methods used to achieve the aforementioned purposes. Moreover, when dealing with modified drug release vaccines for nasal administration, CS, SA, and NPs can be used to improve drug transport. 

## Figures and Tables

**Table 1:** Advantages and disadvantages of each route of administration.

Route (Absorption Site)	Advantages	Disadvantages	Barrier Properties and Delivery Challenges
Intravenous	100% bioavailability Reproducibility	Painful injection Requires medical personnel	None
Subcutaneous	Self-administered Avoids first-pass metabolism	Painful injection	Extracellular Matrix Limited space for injectable volumes
Inhalation (lungs)	Large surface area of absorption Rapid absorption Non-invasive Avoids first-pass metabolism	Variability in dosing can depend on inhaler technique, requiring patient training	Airways transport Surfactant Mucus Epithelial cells Macrophages
Oral (intestines)	Non-invasive Self-administered	Harsh chemical environment Degraded by first-pass metabolism	Epithelial cells Mucus Bacteria Gastrointestinal transit time Acid, enzymes, and proteases
Transdermal (skin)	Non-invasive Self-administered Avoids first-pass metabolism	Major transport barriers Slow absorption	Stratum corneum Constant cell shedding Lipid bilayers that surround corneocytes
Nasal (nasal mucosa surface)	Non-invasive Self-administered Rapid absorption Avoids first-pass metabolism	Low surface area for absorption, which limits total dose Prone to cause irritation	Mucus Epithelial cells
Buccal (oral mucosal surface)	Non-invasive Self-administered Rapid absorption Avoids first-pass metabolism	Low surface area for absorption, which limits total dose Prone to cause irritation	Mucus Epithelial cells
